# Health impact of external funding for HIV, tuberculosis and malaria: systematic review

**DOI:** 10.1093/heapol/czt051

**Published:** 2013-08-05

**Authors:** Thyra E de Jongh, Joanne H Harnmeijer, Rifat Atun, Eline L Korenromp, Jinkou Zhao, John Puvimanasinghe, Rob Baltussen

**Affiliations:** ^1^Gephyra IHC, Laing’s Nekstraat 39-1, 1092 GT, Amsterdam, The Netherlands, ^2^ETC Crystal, PO Box 64, 3830 AB, Leusden, The Netherlands, ^3^Imperial College Business School, Imperial College London, London, UK, ^4^Department of Public Health, Erasmus MC, University Medical Centre, Rotterdam, The Netherlands, ^5^Jiangsu Provincial Center for Disease Control and Prevention, Nanjing, China, ^6^The Global Fund to Fight Against AIDS, Tuberculosis and Malaria, Geneva, Switzerland and ^7^Department of Primary and Community Care, Radboud University Nijmegen Medical Centre, Nijmegen, The Netherlands

**Keywords:** Health financing, donors, developing countries, health outcomes, impact, Africa, Asia

## Abstract

**Background** Since 2002, development assistance for health has substantially increased, especially investments for HIV, tuberculosis (TB) and malaria control. We undertook a systematic review to assess and synthesize the existing evidence in the scientific literature on the health impacts of these investments.

**Methods and findings** We systematically searched databases for peer-reviewed and grey literature, using tailored search strategies. We screened studies for study design and relevance, using predefined inclusion criteria, and selected those that enabled us to link health outcomes or impact to increased external funding. For all included studies, we recorded dataset and study characteristics, health outcomes and impacts. We analysed the data using a causal-chain framework to develop a narrative summary of the published evidence.

Thirteen articles, representing 11 individual studies set in Africa and Asia reporting impacts on HIV, tuberculosis and malaria, met the inclusion criteria. Only two of these studies documented the entire causal-chain spanning from funding to programme scale-up, to outputs, outcomes and impacts. Nonetheless, overall we find a positive correlation between consecutive steps in the causal chain, suggesting that external funds for HIV, tuberculosis and malaria programmes contributed to improved health outcomes and impact.

**Conclusions** Despite the large number of supported programmes worldwide and despite an abundance of published studies on HIV, TB and malaria control, we identified very few eligible studies that adequately demonstrated the full process by which external funding has been translated to health impact. Most of these studies did not move beyond demonstrating statistical association, as opposed to contribution or causation. We thus recommend that funding organizations and researchers increase the emphasis on ensuring data capture along the causal pathway to demonstrate effect and contribution of external financing. The findings of these comprehensive and rigorously conducted impact evaluations should also be made publicly accessible.

**KEY MESSAGES**

- We found very few studies that adequately demonstrate the full process by which external funding has been translated to health impact. Most studies do not move beyond statistical association, as opposed to contribution or causation.
- Funding organizations and researchers should increase the emphasis on ensuring data capture along the causal pathway to demonstrate the effect of external financing.

## Introduction

Between 2002 and 2012 global health initiatives, bilateral and multilateral donor agencies, in particular the Global Fund to fight AIDS, tuberculosis and malaria (Global Fund), the World Bank, and the United States President’s Emergency Plan For AIDS Relief (PEPFAR) and President’s Malaria Initiative (PMI), together with low- and middle-income countries (LMIC), have provided considerable resources to enable the scale-up of disease control programmes for HIV, tuberculosis (TB) and malaria [Institute for Health Metrics and Evaluation 2011; Katz *et al.* 2011; The Global Fund 2011; The United States President’s Emergency Plan for AIDS Relief (PEPFAR) 2011]. These investment decisions are generally guided by an ambition to invest in evidence-based strategies with proven effectiveness. Yet, to date, few studies have systematically assessed or synthesized the evidence as to whether these new additional funds have contributed to health impacts beyond those that might have been achieved without external assistance: evidence is needed to demonstrate whether these investments provide value for money and to guide investment decisions by funding agencies, particularly in a time where the effectiveness of development assistance for health is widely being questioned (Leach-Kemon *et al.* 2012).

We conducted a systematic review of studies published in the peer-reviewed and grey literature to assess the extent to which increased external funding for HIV, malaria and TB control programmes in LMIC has translated into increased service coverage and health impact, and analysed these using a causal-chain framework. Our review spans the period 2003–11, reflecting the time when such external funding for the three diseases substantially increased (Institute for Health Metrics and Evaluation 2011).

## Methods

We developed a study protocol, detailing the search strategy, inclusion criteria, outcomes of interest and analytical methods, using guidance from the Cochrane Collaboration to generate a comprehensive and standardized evidence summary. This protocol was used internally to guide discussions between the researchers and the Global Fund, which had commissioned the study, but was not submitted to any trial registers.

### Search strategies and screening

Between October 2011 and May 2012, we searched PubMed, the Cochrane Library, the World Health Organization (WHO) Global Health Library (regional indexes, WHO Library Information System), the 3IE Systematic Review Database, and databases from the World Bank, the Canadian Evaluation Society, OpenGrey, Kaiser Family Foundation, and the Center for Global Development. Where possible, we used combinations of text words and thesaurus terms such as ‘programme evaluation’ [MeSH], ‘outcome assessment (Health Care)’ [MeSH], ‘financing, organized’ [MeSH], ‘financial support’, HIV, tuberculosis and malaria. Search strategies were tailored to the specific databases. For PubMed in particular, we focused on compiling a comprehensive list of relevant MeSH terms, as these reduced the need for extensive usage of synonyms to search titles or abstracts. The search strategy was tested for robustness by checking whether known articles of relevance were retrieved this way, and by cross-referencing against various alternative search terms. A detailed outline of each of the search strategies used is provided in Annex 1.

Thus identified studies of all designs that provided data on both an intervention group and a comparison group were eligible for inclusion. The comparison could be either to a baseline situation or—preferably—to a concurrent control group that did not receive the intervention. We placed no restrictions on eligible units of analysis. Studies were excluded if they were policy reviews, opinions, editorials, letters to the editor, commentaries, conference abstracts, or if they did not contain relevant quantitative data.

Within this set of eligible studies, we considered all studies reporting effects of external funding for HIV, tuberculosis or malaria control on (1) intervention coverage, (2) quality and responsiveness of services, or (3) health outcomes (e.g. morbidity, mortality, incidence, prevalence). We also looked for outcomes related to potential harms or adverse effects of external financing. We considered all external funding for health, regardless of the amount or the type of health activities supported. It was not essential for a study to specify the amount of funding, but it was critical that programme scale-up or introduction of new activities could be linked to a change in resource availability from external sources. We excluded studies on programmes that were funded solely from national resources. We only considered studies published after 2002 (2003–11), in English or French language, and excluded all duplicate references.

Two reviewers (TdJ and JH) independently screened all retrieved titles (κ = 0.73) and abstracts (κ = 0.70) to determine if studies met the inclusion criteria. We retrieved the full text of all selected articles. The two reviewers then independently assessed their eligibility for inclusion, with a third author (RB) acting as an arbiter to resolve disagreements. We also reviewed references of each article to identify other potentially relevant titles that were then evaluated the same way.

### Data extraction and synthesis

For each study, one of the reviewers (TdJ or JH) extracted the relevant data to a standardized form. This included data regarding the study characteristics (study setting, design, study period, inclusion and exclusion criteria), external funding (funding agency, amount, disbursement period), recipients of the funding (name, type of organization, type and number of beneficiaries), the activities funded (type, quantity), characteristics of the baseline and control group, outcomes and potential confounders. The information was then verified or, if necessary, revised by the second reviewer (JH or TdJ). Any disagreements were resolved through discussion. Because of the wide heterogeneity in study designs, research questions and outcomes reported, in addition to reporting on risk of bias based on methodological quality criteria, we provide a brief qualitative assessment of each included study, discussing the main methodological challenges, assumptions and potential sources of bias (Annex 2).

**Figure 1:**
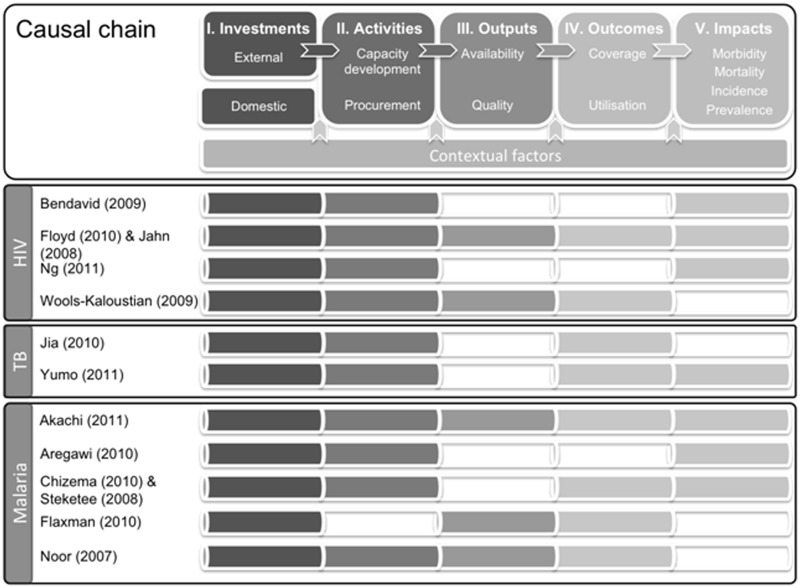
Causal-chain framework, showing the temporal and logical effects of programme investments on health impacts, and the causal-chain elements for which data were provided in each of the included studies. If an element has been reported, this has been indicated by highlighting the corresponding segment in the same shade as the element shown in the framework. Elements that were not reported have been left unshaded. *Note*: Based on the Global Fund 5-year evaluation study framework (Macro International Inc. 2009)

We present data from the included studies in a summary table. As we did not consider a meta-analysis of this heterogeneous set of studies feasible, to present the results we used a narrative summary structured around an analytical framework that uses a causal-chain approach to examine the evidence along a chain linking financial input, via activities, outputs and outcomes, to health impacts (Figure 1, top panel). Any quantitative measures are reported in the same format as in the original studies.

### Quality appraisal

We have assessed the risk of bias in the included studies using a set of criteria derived from grading systems, such as GRADE, which focus primarily on data collection and analysis methods (Atkins *et al.* 2004). However, since all of the included studies used non-randomized designs, and most lacked concurrent controls—relying on historic comparators or regression modelling techniques instead—this type of assessment is of limited value. Therefore, we have additionally assessed the included studies on criteria such as plausibility, strength and specificity of association, consistency of findings, and coherence of the evidence to appraise our findings (Hill 1965; Steketee and Campbell 2010).

## Results

**Figure 2:**
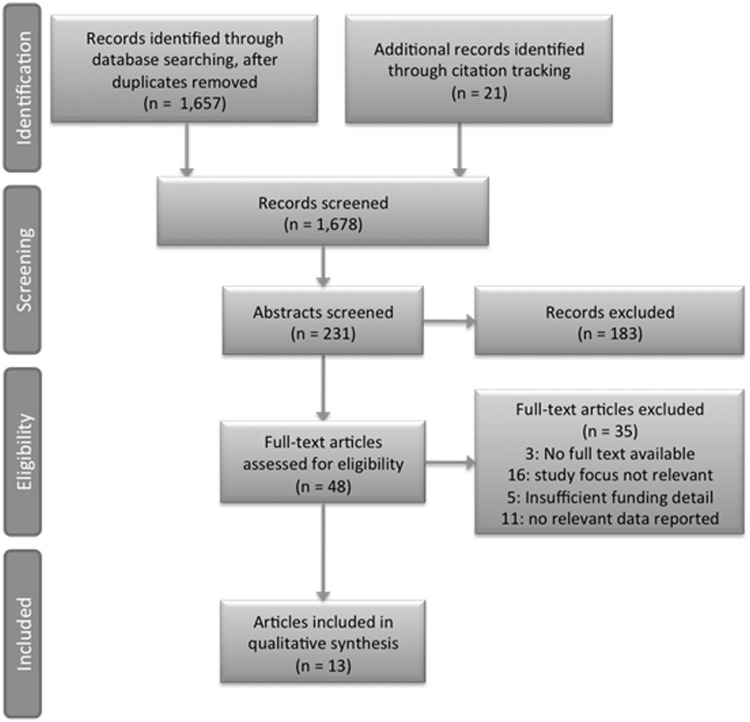
Flow chart showing selection process for included studies

Of the 1657 records retrieved from the searched databases, 210 were retained for further scrutiny. Detailed inspection of their abstracts produced 46 articles for full text screening (Figure 2). We initially selected 12 articles for inclusion. The additional 21 titles were retrieved through citation tracking and evaluated to yield one further article; bringing the total number of included articles to 13, representing 11 individual studies (Figure 2).

### Characteristics of the included studies

Each of the 11 included studies described impacts on a single disease domain: four on HIV, two on tuberculosis and five on malaria. Nine studies were in Africa (including Kenya, Zambia, Cameroon, Zanzibar, Malawi) and two in Asia (India, China). Most studies described country-level programmes, but three studies (Akachi and Atun 2011; Bendavid and Bhattacharya 2009; Flaxman *et al.* 2010) analysed findings from across the African continent. Only the studies by Bendavid and Bhattacharya (2009) and Jia *et al.* (2010) used controlled, albeit non-random, study designs. The others followed various non-controlled designs or were modelling studies. The studies reported on a number of different outcome and impact measures (Tables 1 and 2).

**Table 1:** Summary of health outcomes and impact measures reported in included studies

Indicator category	Indicator	Studies reporting indicator
Service coverage	ART coverage	Floyd *et al.* (2010), Jahn *et al.* (2008)
	TB case detection and notification rates	Jia *et al.* (2010), Yumo *et al.* (2011)
	% of households owning at least one (long lasting) insecticide treated net	Akachi and Atun (2011), Chizema-Kawesha *et al.* (2010) and Steketee *et al.* (2008), Flaxman *et al.* (2010), Noor *et al.* (2007)
	% of pregnant women receiving at least two doses of Intermittent Preventive Therapy	Chizema-Kawesha *et al.* (2010) and Steketee *et al.* (2008)
Service utilization	% of children under-5 years sleeping under an insecticide-treated bed net	Noor *et al.* (2007), Flaxman *et al.* (2010), Steketee *et al.* (2008)
	ART adherence and loss to follow-up rates	Wools-Kaloustian *et al.* (2009)
Health impacts	HIV infections averted	Ng *et al.* (2011)
	HIV prevalence	Bendavid and Bhattacharya (2009), Ng *et al.* (2011)
	Deaths due to HIV/AIDS	Bendavid and Bhattacharya (2009), Floyd *et al.* (2010)
	TB treatment outcomes	Yumo *et al.* (2011)
	Malaria cases	Aregawi *et al.* (2011), Chizema-Kawesha *et al.* (2010)
	Anaemia cases	Aregawi *et al.* (2011), Chizema-Kawesha *et al.* (2010)
	Malaria-attributed mortality	Akachi and Atun (2011), Aregawi *et al.* (2011), Chizema-Kawesha *et al.* (2010)
	All-cause adult mortality	Floyd *et al.* (2010)
	All-cause under-5 mortality	Chizema-Kawesha *et al.* (2010)
	Lives saved	Akachi and Atun (2011)

**Table 2:** Summary and key findings of included studies

Study (year)	Study design	Study setting	Evaluation period(s)	Funding source	Funded activities	Key findings
**HIV**						
Bendavid and Bhattacharya (2009)	Controlled before–after study	Africa; 41 countries	1997–2002 vs 2004–07	PEPFAR	HIV prevention and treatment, infrastructure	• The annual change in HIV-related deaths was lower by 10.5% in PEPFAR countries compared with controls (95% CI: −16.6% to −4.4%, *P* = 0·001). • There was no significant difference in the annual growth in the number of PLHIV, nor in the change in HIV prevalence over the evaluation period, between PEPFAR focus countries and controls.

Floyd *et al.* (2010) and Jahn *et al.* (2008)	Time series study	Malawi; 1 district	2002–08	GF	ART in a ‘public health’ approach	After introduction of a GF HIV grant, in June 2005: • All-cause mortality (15–59 years), which was 10.2 per 1000 person-years in the pre-ART period (2002–05), fell by 16% in ART period 1 (2005–06) and by 32% in ART period 2 (2006–08). • AIDS mortality rate fell from 6.4 per 1000 person-years in the pre-ART period to 4.6 in ART period 1 and to 2.7 in ART period 2. • There was little change over time in non-AIDS mortality. • Treatment coverage among individuals eligible to start ART reached 70% in 2008.

Ng *et al.* (2011)	Observational study, with regression modelling	India; 6 states	2003–08	Bill and Melinda Gates Foundation (BMGF)	HIV prevention in high-risk groups, especially sex workers	• The programme was associated with reductions in HIV prevalence from 2003 to 2008 in all six states, ranging from 12.7% to 2.4% decline. • In three out of six states the amount of grant per PLHIV was significantly associated with lower HIV prevalence among the adult population, based on extrapolation from antenatal clinic data. • The estimated 100 178 HIV infections were averted between 2003 and 2008 as a result of the programme.

Wools-Kaloustian *et al.* (2009)	Cohort study, retrospective	Kenya; 17 health facilities	2001–06	PEPFAR	ART	With the support of PEPFAR funding: • Mean monthly ART enrolment increased from 64 to 815 patients. • Patients enrolled in treatment in progressively earlier states of HIV disease, as indicated by increasing median CD4 cell count (*P* < 0.001) and earlier WHO clinical stage (*P* < 0.001) at time of enrolment. • Time from enrolment to treatment initiation decreased (*P* < 0.001).
**TB**						
Jia *et al.* (2010)	Observational study, with regression modelling	China; 3492 counties	2001–08	GF, World Bank	All aspects of TB control inc. TB drugs, lab strengthening, training, computers, and vehicles), health promotion materials	• From 2002 to 2005 case notification increased in all areas, and then stabilized or began to fall. The increases were much bigger in areas supported by the World Bank (236%) and Global Fund (224%) than in areas that only received Chinese government support (65%). • The average cost per TB case notified was similar in World Bank, Global Fund, and Chinese government areas.

Yumo *et al.* (2011)	Cohort study, retrospective	Cameroon; 1 district hospital	2003–08	GF	Training in DOTS, lab strengthening, waiver of TB treatment and HIV testing fee	Compared with Phase A (2003–05), after introduction of a Global Fund grant in Phase B (2006–08): • Case notification (all TB forms) increased from 102 to 191. • Case detection (new smear-positive cases) increased from 28.3% to 41.6%. • Treatment success rate (all forms) increased from 75.3% to 89.2%. • Default and mortality rates dropped to zero from maximum values (over 2003–05) of 15% and 23%, respectively.

**Malaria**						
Akachi and Atun (2011)	Regression modelling study	Sub-Saharan Africa; 34 countries	2002–08	GF, others	ITNs/LLINs, IRS	As Official Development Assistance for malaria control increased in the period 2002–08: • Distribution of ITNs increased from 9.7 million in 2002 to 46 million in 2008. • Average ITN/IRS household ownership increased from 8.3% to 33%. • Cumulatively, the estimated 237 971 lives (among children under 5) were saved due to ITN/IRS coverage increase.

Aregawi *et al.* (2011)	Time series study	Zanzibar; 6 health facilities	1999–2003 vs 2008	GF, PMI, others	ACT, ITNs/LLINs, IRS, IPTp	After programme scale-up: • Malaria deaths decreased by 90%, malaria in-patient cases by 78% and confirmed malaria out-patient cases by 99·5% (all *P* < 0.025). • Among children under-5, anaemia in-patient cases decreased by 85% (*P* < 0.025); anaemia deaths and out-patient cases declined without reaching statistical significance. • In children under-5, the proportion of all-cause deaths due to malaria fell from 46% to 12% (*P* < 0.01); those due to anaemia from 26% to 4% (*P* < 0.01).
Chizema-Kawesha *et al.* (2010) and Steketee et al. (2008)	Case study	Zambia; country	2000–08	GF, USAID/PMI, BMGF, Japan International Cooperation Agency and others	ACT, ITNs/LLINs, IRS, IPTp, Rapid Diagnostic Testing	• Household ITN ownership tripled between 2001 and 2006, with 44% of households owning at least one ITN in 2006. ITN use doubled between 2004 and 2006, achieving use levels of 23% in young children and 24% in pregnant women. After programme scale-up (2006–08): • Increasing ITN coverage was associated in time and location with reductions in parasitaemia (53%) and severe anaemia (68%) in children under-5. • 62% of pregnant women received at least two doses of IPTp vs 54% before scale-up. • Under-5 mortality fell from 168 to 119 per 1000 live births.

Flaxman *et al.* (2010)	Regression modelling study	Africa; 44 countries	1999–2008	All external funding for malaria	ITNs	Excluding four outlier countries, each US$1 per capita in malaria development assistance for health was associated with an increase in ITN household ownership coverage (5.3% points) and ITN use in children under-5 (4.6% points).

Noor *et al.* (2007)	Cohort study, prospective	Kenya; 4 districts	2004–06	GF, DFID	ITNs/LLINs	• The proportion of children under-5 who slept under a recently treated ITN in the previous night increased from 7.1% in 2004, to 23.5% in 2005 (after introduction of heavily subsidized nets), and to 67.3% in 2006 (after introduction of free mass distributed ITNs). • Socioeconomic inequity in net coverage progressively decreased.

*Note*: ACT = artemisinin-based combination therapy; ART = anti-retroviral therapy; IPTp = intermittent preventive therapy in pregnancy; IRS = indoor residual spraying; ITN = insecticide-treated net; LLIN = long-lasting insecticidal net; PLHIV = person living with HIV.

Studies on HIV evaluated scaling up of anti-retroviral treatment (ART) in Kenya (Wools-Kaloustian *et al.* 2009) and Malawi (Floyd *et al.* 2010; Jahn *et al.* 2008), of prevention activities in India (Ng *et al.* 2011), and PEPFAR support for combined prevention and treatment programmes in 41 countries (Bendavid and Bhattacharya 2009). In the two included studies that analysed TB control (Jia *et al.* 2010; Yumo *et al.* 2011), external funding was used primarily for strengthening capacity in diagnosis and treatment of TB in Cameroon and China, respectively. The impact of external funding for scaling-up malaria control programmes, centred largely on distribution of insecticide-treated nets and scale-up of artemisinin-based combination therapy, was assessed in Kenya (Noor *et al.* 2007), Zanzibar (Aregawi *et al.* 2011), and Zambia (Chizema-Kawesha *et al.* 2010; Steketee *et al.* 2008), while two studies (Akachi and Atun 2011; Flaxman *et al.* 2010) compared data from across 44 and 34 African countries, respectively.

### Constructing the causal chain

The included studies varied greatly in the total amount of external funding involved, ranging from a single grant worth US$5.8 million (Yumo *et al.* 2011) to cumulative PEPFAR spending over 5 years (2003–08) and 12 countries worth US$15 billion (Bendavid and Bhattacharya 2009) (Table 3). Studies could not be directly compared on the basis of per capita expenditure, because either the number of beneficiaries of the intervention was not given or it was not clear how the funds had been allocated over the project lifetime or the study period.

Three studies assessed individual interventions, namely ART (Floyd *et al.* 2010; Jahn *et al.* 2008; Wools-Kaloustian *et al.* 2009) or insecticide-treated bed nets (Noor *et al.* 2007). Seven others evaluated a more comprehensive package of prevention, care and treatment activities, sometimes including health system strengthening activities such as development of laboratories or staff capacity (Akachi and Atun 2011; Aregawi *et al.* 2011; Bendavid and Bhattacharya 2009; Chizema-Kawesha *et al.* 2010; Jia *et al.* 2010; Ng *et al.* 2011; Steketee *et al.* 2008; Yumo *et al.* 2011). In one study (Flaxman *et al.* 2010), development assistance for malaria was not further disaggregated into specific components. All studies found that the increased availability of external funds resulted in a degree of programme scale-up or in the introduction of new activities. However, most did not provide quantitative data on either the scope of activities implemented or on the amount of funding involved. Across the 11 studies, health outcomes and impact were assessed in a period of less than 1 year to 7 years after the initial scale-up in funding. It was not possible to characterize an average time lag from funding to impact more precisely, as many studies lacked detailed data on funding patterns over time, and where specified, the funding increase was mostly gradual and continuing into the period of impact evaluation. It should also be noted that sometimes the increases in external funding co-occurred with increases in domestic spending (Chizema-Kawesha *et al.* 2010; Jia *et al.* 2010; Ng *et al.* 2011; Steketee *et al.* 2008) or were additional to other, pre-existing external funding (Aregawi *et al.* 2011; Bendavid and Bhattacharya 2009; Jia *et al.* 2010).

Eight studies (Akachi and Atun 2011; Chizema-Kawesha *et al.* 2010; Flaxman *et al.* 2010; Jahn *et al.* 2008; Jia *et al.* 2010; Noor *et al.* 2007; Steketee *et al.* 2008; Wools-Kaloustian *et al.* 2009; Yumo *et al.* 2011) reported the effect of programme scale-up on service availability and coverage, and subsequently on service utilization. Programme investments were found to be associated with increased access and adherence to ART treatment (Wools-Kaloustian *et al.* 2009); increased coverage and utilization of bed nets (Akachi and Atun 2011; Flaxman *et al.* 2010; Noor *et al.* 2007; Steketee *et al.* 2008); increased coverage of indoor residual spraying, intermittent preventive therapy in pregnancy, and artemisinin-combination therapy (Steketee *et al.* 2008); and with increased TB case notification and detection (Jia *et al.* 2010; Yumo *et al.* 2011) (Table 2).

**Table 3:** Summary of funding data reported in included studies

Study	External funding		Other funding		Funding period^b^
	Source(s)	Amount^a^	Source(s)	Amount	
**HIV**					
Bendavid and Bhattacharya (2009)	PEPFAR	US$ 15b (US$6 billion disbursed by 2007)	Governments, GF and other donors	Not specified	2003–08
Floyd *et al.* (2010) and Jahn *et al.* (2008)	GF	Not specified	Not specified		2004–?
Ng *et al.* (2011)	Bill and Melinda Gates Foundation	US$ 258m	Government	US$460 million for phase 2 (1999–2006); US$ 2.5b for phase 3 (2007–12), of which two-thirds allocated for HIV prevention.	2003–08
Wools-Kaloustian *et al.* (2009)	PEPFAR	US$ 6.5m + free ARVs	Not specified		2004–?
**TB**					
Jia *et al.* (2010)	GF, WB, others	WB + others US$ 432m; GF US$ 151m^c^	Central and local government	US$ 159m	2001–08
Yumo *et al.* (2011)	GF	US$ 5.8m	Government	Not specified	2005–09
**Malaria**					
Akachi and Atun (2011)	All development assistance for malaria control, including GF	US$ 1.9b, of which GF US$ 1.4b (as disbursed by 2008)	Not specified		2002–08
Aregawi *et al.* (2011)	GF, PMI, others	Not specified	Government	Not specified	2003–?
Chizema-Kawesha *et al.* (2010) and Steketee *et al.* (2008)	GF, USAID/PMI, WB, and others	US$ 130m	Government	Not specified	2003–08
Flaxman *et al.* (2010)	All development assistance for malaria control	Not specified	Not specified		2000–08
Noor *et al.* (2007)	DFID, GF	US$ 106m (DFID US$ 56m; GF US$ 17m)	Not specified		2004–?

*Notes*: ^a^The amounts reported are those that were *allocated* in the form of grants or donor pledges, unless specified otherwise. ARV = antiretrovirals; GF = The Global Fund to Fight AIDS, Tuberculosis and Malaria; PMI = The President’s Malaria Initiative; USAID = The United States Agency for International Development; WB = The World Bank.

^b^The funding period refers principally to the period over which the *external* funding was provided. If the funding period over which stated other funding was provided differs, this has been indicated separately. If the included study only mentioned a starting year for the funding, without additional information regarding the duration of the funding period, this has been indicated by presenting the end year with?

^c^Amounts converted from Chinese Yen, using the conversion factor provided by Jia *et al.*

Health impact was reported in 6 of the 11 studies, which showed that programme scale-up was associated with reductions in HIV-related mortality (Bendavid and Bhattacharya 2009; Floyd *et al.* 2010; Jahn *et al.* 2008), malaria-related morbidity and mortality (Akachi and Atun 2011; Aregawi *et al.* 2011; Chizema-Kawesha *et al.* 2010; Steketee *et al.* 2008), lives saved through ITN/IRS coverage (Akachi and Atun 2011), and improved TB treatment outcomes (Yumo *et al.* 2011) (Table 2). Studies of HIV funding, however, found only limited impact on the number of new HIV infections Ng *et al.* (2011) and no statistically significant effect on adult HIV prevalence or changes in the number of people living with HIV (Bendavid and Bhattacharya 2009). Although Ng *et al.* showed that an HIV prevention project implemented in six states in India was associated with reductions in the number of new HIV infections compared with an estimated counterfactual, there was substantial variation between states in the effect sizes (Ng *et al.* 2011).

### Quality of the studies included

All included studies showed a high risk of bias (Annex 2). As none of the studies were randomized trials, none used any method of randomization or concealment of allocation. Only two studies included a form of control group (Bendavid and Bhattacharya 2009; Jia *et al.* 2010); in neither of these was the control group adequately matched to the intervention group, although the study by Bendavid and Bjattacharya attempted to correct for differences in baseline characteristics during analysis. In none of the studies that used controls, it was clear whether blinding of outcome assessors or analysts took place. It was not possible to judge whether any selective outcome reporting took place, as we did not retrieve any study protocols.

A number of additional quality issues were evident in the studies included in the review. The first of these relates to incomplete or weak data sets used by the studies, as explicitly acknowledged by some study authors, which meant study findings had to be interpreted with caution (Aregawi *et al.* 2011; Chizema-Kawesha *et al.* 2010; Jia *et al.* 2010). In order to, at least partially, overcome the problem of weak data sets, two of the studies strengthened the evidence for impact of malaria control activities on malaria-attributed morbidity and mortality by providing corroborating data on associated parameters such as parasitaemia and anaemia prevalence (Aregawi *et al.* 2011; Chizema-Kawesha *et al.* 2010; Steketee *et al.* 2008).

The second issue relates to attribution of observed impacts to the intervention according to our framework. Although the causal-chain framework outlines a logical path from investments to outcomes and impact, this may be an over-simplification in cases where there is progressive implementation of a package of activities. For instance, in two of the included studies (Aregawi *et al.* 2011; Chizema-Kawesha *et al.* 2010) the malaria disease burden was already declining before programmes had been implemented in their entirety—declines attributed to one or more elements introduced before the remainder of the package. In some studies the time between the intervention and the assessment may have been too short to observe health impacts, but reported improvements in intermediate output and outcome indicators have helped build a case for plausible contribution of investments to improved impacts. A particular challenge is that most of the included studies were not experimental designs with proper controls. Thus, it is difficult to rule out that the observed effects would not have occurred without the external investments. Three of the included studies (Akachi and Atun 2011; Aregawi *et al.* 2011; Ng *et al.* 2011) attempted to tackle this design problem by comparing findings against hypothetical ‘counterfactuals’ obtained through modelling. The validity of these approaches, however, rests on the quality of the data, robustness of the models employed, the suitability of the counterfactual used and the accuracy of the input variables, all of which are difficult to independently corroborate.

Third, a valuable indication of the validity of the overall evidence relates to the strength of association between the intervention and the observed effect size. Particularly in the HIV studies, some of the observed effects were relatively small, whilst other effects were not statistically significant (Bendavid and Bhattacharya 2009; Floyd *et al.* 2010; Jahn *et al.* 2008; Ng *et al.* 2011). The strength of the evidence for a causal impact of the programme interventions is strongest when there is a demonstrable ‘dose–effect’ relationship between the intensity of services and the observed impacts. Three studies indeed observed an association between the amount of investment and the magnitude of impact on HIV prevalence (Ng *et al.* 2011), the number of lives saved due to ITN/IRS coverage (Akachi and Atun 2011), or the number of TB cases reported (Jia *et al.* 2010), respectively.

## Discussion

Whilst the included studies are heterogeneous in the types and magnitudes of the reported effects, settings and sample sizes, their findings consistently point to improved health outcomes and impacts. The combined evidence across all included studies therefore suggests that in the period studied external funding for HIV, TB and malaria control has contributed to reductions in morbidity and mortality from these three major diseases. However, this evidence is derived from only a small set of studies that were identified in the peer-reviewed literature and it is thus difficult to draw conclusions about impacts more generally. Furthermore, only two of the included studies (Akachi and Atun 2011; Floyd *et al.* 2010; Jahn *et al.* 2008) provided evidence on the entire casual chain—from investments to activities, outputs, outcomes and impact—and were hence able to directly link impact to external funding. All others fell short of reporting to what extent activities were scaled up, what outputs were generated, or what outcomes were achieved as a result of financing, and could thus only show statistical association. This paucity of scientifically rigorous, published studies to generate evidence is remarkable, considering the US$ 202 billion spent over the period 2002–11 on Development Assistance for Health (Institute for Health Metrics and Evaluation 2011).

Whilst this review finds that in several of the 11 included studies, overall external funding in HIV, TB and malaria programmes was associated with improved health impact, with contribution demonstrated in some, questions about the sustainability of these health impacts remain unanswered. One study included in the review (Jia *et al.* 2010) showed how with reduced external funding programme efforts diminished, resulting in a worsening of health outcomes. Others have argued that continued funding is needed to sustain or exceed current levels of impact, albeit without underpinning evidence to support such arguments (Noor *et al.* 2007; Wools-Kaloustian *et al.* 2009). Although these claims seem convincing at face value, as yet, they lack a strong empirical basis.

The studies included in the review provide limited evidence of whether external funding has increased accessibility to services, particularly for underprivileged and vulnerable groups. Only four studies explored the equity dimension of investments, with three (Chizema-Kawesha *et al.* 2010; Jia *et al.* 2010; Noor *et al.* 2007) showing positive benefits of overcoming inequitable service provision. This potential for global health initiatives to produce greater equity in access to health services has been noted previously (Hanefeld 2008), but further assessment is required to demonstrate realization of this potential.

It is worth noting that none of the included studies reported on adverse effects from the external funding nor was the potential for such effects discussed beyond the aforementioned concerns regarding programme sustainability. Known examples of adverse effects include health worker migration from other programmes into the externally funded programmes, or diminished focus on other health services leading to a worsening of health outcomes in these areas.

### Limitations of this review

Our conclusions are based on only 11 studies, which are unlikely to be representative of all externally funded programmes in over 150 countries (The Global Fund 2012) for several reasons. First, for the objective of this review it was essential that studies explicitly reported the role of external funding in programme scale-up so that any observed impacts could be linked to changes in external funding. Consequently, studies that did not explicitly describe underlying funding changes or did not link funding changes to health outcomes had to be excluded, even if external funding was likely or known to have enabled the documented programme scale-up. For example, programme scale-up in impact assessments by Otten *et al.* (2009) and Reniers *et al.* (2009) was likely enabled by external funds, but this was not made explicit and so we excluded these studies. Likewise, whilst studies by Sharp *et al.* (2007) and by Steketee and Campbell (2010) did refer to increased external funding, outcome measures were not temporally linked to these funding changes so that it was not possible to determine impact from this funding. Second, whilst we have aimed to identify all relevant studies, our search methodology presents some limitations. As in any systematic search, we may have missed relevant studies if search terms were not present in the MeSH terms, title or abstract of the article. Moreover, we could not take into account the large number of evaluations, country reviews and management reports that are used by programmes or national governments themselves, since most of these are not available or indexed in searchable, open-access databases. Contacting the major funding agencies directly to request this documentation was discussed, but ultimately not pursued because of the unsystematic nature of such a strategy, which could have introduced additional sources of bias. These concerns are amplified by the fact that the evaluation literature is likely to show a publication bias towards successful interventions (Dwan *et al.* 2008; Song *et al.* 2009). Particularly when studies are directly commissioned or funded by organizations with a vested interest, for instance to advocate for continued contributions from donors, caution should be exercised when interpreting the evidence base. Third, some of the 11 studies included present findings from small-scale projects with no more than a few hundred beneficiaries and relatively small amounts of external funding, and as such may not present an accurate reflection of the true scope of all externally funded programmes.

An additional consideration in interpreting the evidence from this review is that we have taken a rather simplistic, linear view of the causal chain between external investments and impacts. In reality, this pathway may be affected by a multitude of contextual factors that can act to either enforce or counter the direction of effect. Yet, most of the included studies are short on the contextual details that can influence programme effectiveness, such as the social acceptability of interventions or the characteristics of the health system in which supported programmes are set (Atun *et al.* 2005; Katz *et al.* 2010; Tkatchenko-Schmidt *et al.* 2010). At worst, it is possible for external funding to produce undesirable side effects (Car *et al.* 2012). For example, this review has not looked at the impact external funding has had on domestic health spending. Whilst external contributions are generally contingent upon additionality, it is conceivable governments’ budget allocation decisions are informed by the anticipated availability of other funds. Consequently, increases in external funding may lead to displacement of government funds, eventually resulting in reduced programme ownership and sustainability (Farag *et al.* 2009; Lu *et al.* 2010). Also impacts of external funding on, among others, governance, health sector efficiency or health worker migration patterns fall beyond the scope of this review, although these are important considerations in policy discussions concerning development assistance for health.

### Implications for policy and research

The narrow base on which the conclusions of this review are founded should not be interpreted to mean that external funds from donors and global health initiatives have had little impact, or that data on positive health benefits of these investments do not exist. What this review highlights, however, is a shortage of studies that are conducted to a rigorous scientific standard, and of which results are made publicly available, to generate much needed evidence on health impact, effectiveness and efficiency of external funding. Even fewer such studies have adequately provided information on all steps along the causal chain. In fact, this lack of comprehensive, robust and reliable data has also been recognized by others as a major problem in the debate on aid effectiveness (Stuckler *et al.* 2012). We recommend wider use of this framework in developing and publishing impact studies, with an emphasis on demonstrating attribution. These studies should include an adequate description of contextual factors impacting at all stages along the causal chain, and be inclusive of reporting potential undesirable effects. This requires a more systemic approach to impact evaluation than one that is focused solely on programme outcomes—ideally also accounting for system-wide effects.

One would expect that as a result of substantial investments in data collection systems for health programmes made over the last decade by major financing institutions, a wealth of rigorously conducted studies would have been published, covering the full sequence from financing, through inputs, outputs and outcomes, to health impact (Shakarishvili *et al.* 2010; The Global Fund 2011). Indeed, in recent years countries and key international financing agencies have been making strides in evaluating their achievements. Between 2006 and 2010, global health initiatives have launched several evaluation strategies and agendas [President's Malaria Initiative 2012; The Global Fund to Fight AIDS Tuberculosis and Malaria 2012; The United States President’s Emergency Plan for AIDS Relief (PEPFAR) 2012], and technical agencies have produced guidelines for standardized national programme reviews and evaluations (UNAIDS 2012; World Health Organization 2009, 2010). Increasing numbers of donor-supported national HIV, TB and malaria programmes are conducting comprehensive programme reviews and evaluations, supported by activities to strengthen essential data collection through routine disease surveillance systems, surveys, data quality assessments and sometimes sentinel sites. However, it has been noted previously that routine evaluation frameworks and policies are frequently not clearly articulated up front*;* rather, decisions on what to evaluate, and when, are often made *ad hoc* and evaluation requirements vary between funding organizations (Kebede *et al.* 2012).

Furthermore, evaluations conducted, or commissioned, by funding agencies vary in the strength of their design and the quality of data and analysis, and are rarely put through a process of scientific peer-review and publication. In itself this is not surprising as this process is generally time-consuming and labour intensive, at odds with the consultancy-driven nature of many programme evaluations, which are conducted to tight deadlines and are aimed primarily at providing rapid, actionable recommendations. There is, in truth, very little incentive for funding agencies to engage in peer-reviewed publishing, which is mainly the domain of academic researchers.

Overall, our findings suggest a need for increased investment in strong country-level comprehensive evaluations, the results of which are to be made publicly accessible and preferably subject to peer-review. First, funding agencies should work to improve reporting of their funding in a standardized and transparent manner, with proper documentation of programme spending by source and service delivery area (Sridhar and Batniji 2008). Furthermore, investments aimed at strengthening country level health information systems should ensure data capture along the full causal pathway to demonstrate effect and contribution of external financing. In these challenging economic times, there is a clear case for donors and countries to support publicly accessible, rigorous and comprehensive programme impact evaluations to demonstrate value for money.

## Financial disclosure

This study was commissioned and supported by The Global Fund to fight AIDS, Tuberculosis and Malaria (http://www.theglobalfund.org/). The funder had no role in study design, data collection and analysis, decision to publish, or preparation of the manuscript.

## Competing Interests

At the time of writing, three auhors (E.K., J.P. and J.Z.) were employees of the Global Fund. The views expressed in this article are those of the authors and do not necessarily represent the decisions, policy or views of the Global Fund to fight AIDS, Tuberculosis and Malaria. R.A. is funded by Imperial College London. The corresponding author had full access to all the data in the study and had final responsibility for the decision to submit for publication.

## Conflict of interest

None declared.
